# Deep-Learning Analysis of Peripheral Blood Smear for Prediction of Acute Inflammatory States and Chronic Comorbidities: Development and Internal Validation Study

**DOI:** 10.2196/100445

**Published:** 2026-11-13

**Authors:** Jaehyeok Jang, Minsu Lee, Yu Rang Park, Jaewoo Song

**Affiliations:** 1 Department of Laboratory Medicine, Yonsei University College of Medicine, Seoul, Republic of Korea, 82 2-2228-2493; 2 Department of Biomedical Systems Informatics, Yonsei University College of Medicine, 50-1 Yonsei-ro, Seodaemun-gu, Seoul, 03722, Republic of Korea, 82 2 2228 2493

**Keywords:** deep learning, peripheral blood smear, systemic states, cell morphology, inflammation, septic shock, comorbidity

## Abstract

**Background:**

Peripheral blood smear (PBS) examination provides morphological information on immune cells that reflects systemic pathophysiology, yet whether it can be used to predict systemic states has not been systematically established.

**Objective:**

This study aimed to develop and evaluate an integrated deep learning framework that leverages cell-level morphology from routine PBS examinations to discriminate acute inflammatory states and chronic comorbidities.

**Methods:**

An integrated deep learning framework was developed for PBS analysis, in which cell-level morphological features were extracted and aggregated into slide-level representations to predict systemic states. A total of 51,369 PBS examinations were used for self-supervised cell-level pretraining and 9211 for slide-level downstream tasks. Outcomes comprised acute inflammatory states and chronic comorbidities. Acute inflammatory states included white blood cell (WBC) count (categorized as low, normal, or high), elevated C-reactive protein (CRP), elevated erythrocyte sedimentation rate (ESR), and septic shock. Chronic comorbidities included the Charlson Comorbidity Index (CCI), cardiovascular disease (CVD), chronic kidney disease (CKD), and diabetes mellitus (DM). Discriminative performance was assessed using the area under the receiver operating characteristic curve (AUROC) with 95% CIs, computed on the test set of the development dataset and on the holdout dataset. The framework was compared with a demographic model based on age and sex and with a cell-type-only model without morphological features. As a secondary analysis, temporal validation was performed using a chronological split.

**Results:**

The framework discriminated acute inflammatory states, achieving development and holdout AUROCs of 0.852 (95% CI 0.836‐0.867) and 0.849 (0.833‐0.864) for WBC count, 0.891 (0.870‐0.913) and 0.896 (0.876‐0.915) for CRP, 0.838 (0.794‐0.878) and 0.844 (0.799‐0.882) for ESR, and 0.849 (0.807‐0.886) and 0.822 (0.783‐0.853) for septic shock. Discrimination of chronic comorbidities was more modest but consistent across the 2 datasets, with AUROCs of 0.792 (0.762‐0.818) and 0.781 (0.751‐0.808) for CCI, 0.696 (0.665‐0.727) and 0.680 (0.654‐0.708) for CVD, 0.724 (0.690‐0.755) and 0.728 (0.696‐0.756) for CKD, and 0.645 (0.610‐0.680) and 0.638 (0.603‐0.674) for DM. The framework significantly outperformed the cell-type-only model for all outcomes in both datasets but did not significantly outperform the demographic model for CVD in the holdout dataset or for DM in either dataset. AUROCs in the temporal holdout dataset ranged from 0.621 for DM to 0.883 for CRP, consistent with those in the temporal development test set (0.666 to 0.889).

**Conclusions:**

An integrated deep learning framework for automated PBS analysis can provide information associated with inflammation-related laboratory results and clinical outcomes, with stronger discrimination for acute inflammatory states than for chronic comorbidities. These exploratory findings suggest the potential of deep learning-based PBS analysis as a novel approach to assessing systemic states in clinical practice.

## Introduction

The peripheral blood smear (PBS) remains an essential diagnostic technique routinely performed in clinical laboratories. Unlike the complete blood count (CBC), which provides blood cell indices from the overall cell population, PBS enables direct cytomorphological assessment of individual blood cells through microscopic examination [1]. Given that the morphological features of blood cells reflect lineage identity, cellular maturation stage, and pathological abnormalities, PBS serves as an indispensable tool for clinical practice, enabling not only differential counting of white blood cells (WBCs) but also diagnosis of hematological disorders and infectious conditions, as well as verification of flagged results from automated hematology analyzers [2,3].

Morphological alterations of WBCs are recognized as valuable indicators across a range of clinical contexts. WBCs, known to be key modulators of systemic inflammatory responses that contribute to the pathophysiology of diverse clinical conditions, exhibit structural properties that reflect functional and subpopulation heterogeneity [4,5]. For example, hematological parameters reflecting structural characteristics of various WBC lineages, including neutrophils, lymphocytes, and monocytes, have been reported to be associated with inflammatory conditions and patient outcomes [6-9]. Furthermore, cytoplasmic changes in neutrophils and reactivity patterns in lymphocytes have been shown to exhibit dynamic variation in response to therapeutic intervention [10]. Stratification of immature neutrophils based on morphological features has demonstrated clinical utility in the assessment of inflammatory status and prognosis [11].

Computational approaches have been extensively developed for PBS analysis, demonstrating remarkable performance in automated leukocyte identification and morphological categorization [12,13]. These deep learning approaches, which utilize morphological analysis of WBCs, have demonstrated clinical utility in the diagnosis and subtyping of hematological malignancies [14-17]. Furthermore, the morphological characteristics of WBCs are closely linked to their functional and activation states, and emerging evidence suggests that morphological features can be leveraged to predict infectious disease [18]. While these findings indicate that deep learning-based PBS analysis holds potential beyond hematological malignancies, its applicability to a broader spectrum of systemic states remains largely unexplored.

In this study, we introduce an integrated deep learning framework for the analysis of PBS examinations, designed to assess systemic states. The framework was evaluated across diverse clinical classification tasks, with outcomes encompassing acute inflammatory states and chronic comorbidities, predefined from laboratory parameters or disease diagnoses.

## Methods

### Ethical Considerations

This study was approved by the Institutional Review Board (IRB) of Severance Hospital, Yonsei University, Seoul, South Korea (4-2025-0453 and 4-2025-1648). The requirement for informed consent was waived by the IRB because this was a retrospective study that involved minimal risk to participants, and all data were anonymized prior to analysis. This study was conducted in accordance with the principles of the Declaration of Helsinki. No compensation was provided to participants, as this study involved only retrospective analysis of existing data. The cell images presented in the paper and Multimedia Appendices are microscopic images of individual blood cells and do not contain any information by which individual participants can be identified.

### PBS Datasets

PBS examinations performed in the clinical laboratory at Severance Hospital, Seoul, South Korea, between 2022 and 2025 were used. The dataset was organized into 2 streams aligned with the modeling objective. PBS examinations collected from 2022 to 2023 served as the pretraining dataset for cell-level analysis, and those collected from April 2024 to December 2025 served as the dataset for slide-level analysis. For the slide-level dataset, only examinations from patients aged 19 years and older were included. This dataset was then split at the patient level, stratified by age and sex, into development and holdout datasets in an 8:2 ratio, independently of the downstream task.

Each PBS examination consisted of multiple individual cell images, with a variable number of cells per examination. All cell images were obtained using the DI-60 automated digital cell morphology analyzer (Sysmex Corporation), which captures red-green-blue cell images at 100 times magnification. For the pretraining dataset, individual cell images were used without slide-level grouping, and the corresponding examinations were not used for any slide-level task. For the slide-level dataset, PBS examinations retained their slide-level structure as sets of constituent cell images.

### Model Development

The framework consisted of 3 independently trained components organized into 2 analyses, a cell-level analysis and a slide-level analysis (Figure 1). The cell-level analysis included a cell encoder, which extracted a feature representation from each cell image, and a cell-type classifier, which assigned a predicted cell-type label to each cell feature. These cell features and predicted cell-type labels, produced by the frozen cell encoder and cell-type classifier, served as the input to the slide-level analysis, in which a type-aware transformer encoder integrated the cells of a single PBS examination into a slide-level representation for systemic state classification.

**Figure 1.**
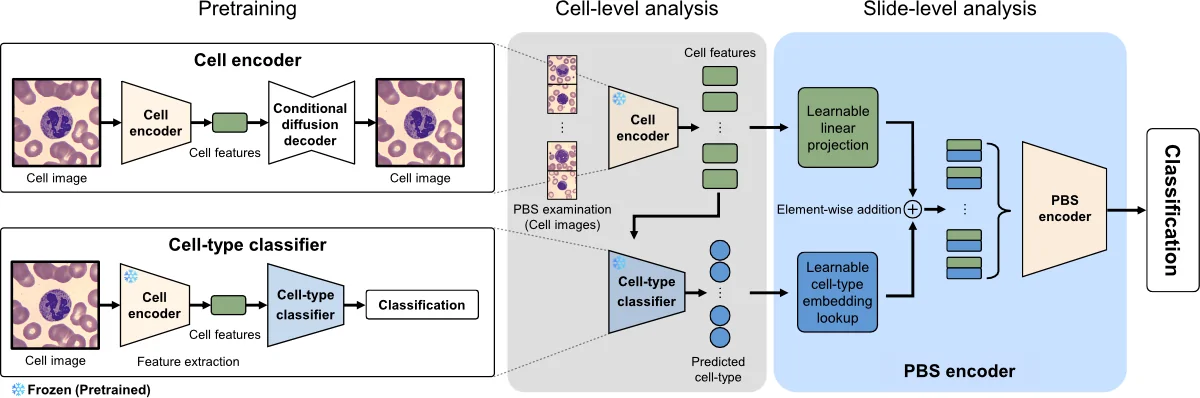
The framework comprises 3 independently trained components. In the pretraining stage, the cell encoder is trained to learn morphological representations from individual cell images, and the cell-type classifier is trained to categorize each cell image into predefined cell types using the extracted cell features. In the slide-level analysis stage, the peripheral blood smear encoder, which aggregates cell-level information, is trained for slide-level task-specific classification. PBS: peripheral blood smear.

The cell encoder employed a diffusion autoencoder architecture, which combined a semantic encoder that produced a latent embedding with a conditional diffusion decoder that reconstructed the input image from Gaussian noise conditioned on that embedding [19]. The encoder was trained in a self-supervised manner on all cell images from the pretraining dataset, and the trained semantic encoder was retained as the cell encoder for downstream use.

The cell-type classifier was a multilayer perceptron that took cell features from the cell encoder as input and predicted the cell type of each image. Cell images were annotated by a laboratory physician as one of 9 categories, comprising neutrophil, lymphocyte, monocyte, eosinophil, basophil, immature cells (promyelocyte, myelocyte, metamyelocyte, and blast cell), nucleated red blood cell (nRBC), platelet, and unidentifiable image. The classifier retained platelet and unidentifiable images as separate output classes, both of which were subsequently treated as noneligible images. A total of 82,000 cell-type annotated images from the pretraining dataset were used for classifier training, partitioned into training and validation sets at a ratio of 8:2. Classification performance was evaluated on 20,000 cell-type annotated images randomly sampled from each of the development and holdout datasets. The predicted cell-type labels for the retained cells were carried forward as auxiliary information for the slide-level model.

The slide-level model was a type-aware transformer encoder operating on the constituent cells of a single PBS examination. Each cell feature was projected into a token embedding through a linear layer, and its predicted cell-type label was mapped to a learnable type embedding via a lookup table. The 2 embeddings were combined by element-wise addition to form the final token for each cell, and PBS examinations with variable numbers of cells were handled by padding and attention masking. The resulting tokens were aggregated via attention pooling into a slide-level representation, which was passed to task-specific classification heads for outcome prediction. Within each PBS examination, cell images predicted as noneligible were excluded, and only nucleated cells comprising WBCs and nRBCs were used as input to the model (hereafter, the base model). In routine manual PBS analysis, 100 WBCs are examined per slide, and nRBCs are reported as the count per 100 WBCs. Accordingly, only PBS examinations containing at least 100 nucleated cells were included in the slide-level analysis [1-3]. To assess the effect of excluding platelet images, we additionally trained a platelet-included model, in which images predicted as platelets were retained as input tokens with their own cell-type embedding and only unidentifiable images were excluded, with all other aspects of the model and training procedure identical to those of the base model described above. For each downstream task, the development dataset was partitioned at the patient level into training, validation, and test sets at a 7:1:2 ratio. All areas under the receiver operating characteristic curve (AUROCs) reported for the development dataset were computed on this test set. The holdout dataset served as an additional test set, applied identically across all tasks for independent evaluation.

### Temporal Validation

As a secondary analysis, the slide-level dataset was repartitioned by time to assess temporal generalizability. The same eligibility criteria as in the main analysis were applied, including an age of 19 years or older, exclusion of noneligible images, and a minimum of 100 nucleated cells per examination. PBS examinations received from April 2024 to September 2025 constituted the temporal development dataset, and those received from October to December 2025 constituted the temporal holdout dataset. To separate the 2 datasets at the patient level, patients included in the temporal development dataset were excluded from the temporal holdout dataset, and their examinations received during the holdout period were not used, so that the 2 datasets shared no patients. For each downstream task, the temporal development dataset was partitioned at the patient level into training, validation, and test sets at a 7:1:2 ratio. Performance was evaluated on the test set of the temporal development dataset and on the temporal holdout dataset.

### Clinical Outcomes for PBS Examination

To evaluate slide-level prediction of systemic states, we prespecified 2 categories of clinical outcomes, namely acute inflammatory states and chronic comorbidities, and assigned task-specific labels independently for each PBS examination. For the acute inflammatory states, the outcomes comprised laboratory indices that are routinely used to assess systemic inflammatory states, along with septic shock as a representative acute clinical condition. Across both categories, clinical outcomes were ascertained from laboratory tests or from clinical diagnoses based on the *International Statistical Classification of Diseases, Tenth Revision (ICD-10)* codes.

Laboratory test-based outcomes were defined using WBC count, C-reactive protein (CRP), and erythrocyte sedimentation rate (ESR). WBC count was categorized according to the reference range (4.0‐10.8×10⁹/L): low (leukopenia), normal, and high (leukocytosis). CRP and ESR were dichotomized into normal versus elevated according to the reference ranges (<8.0 mg/L and <15.0 mm/h, respectively). Test results were obtained from tests performed within one day of the PBS examination, and when multiple values were available, the temporally closest result was selected. PBS examinations without corresponding test results meeting these criteria were excluded.

Septic shock was defined by the presence of sepsis-related *ICD-10* codes (A40, A41, and R57.2) in conjunction with bacterial culture testing and concurrent administration of antibiotics and vasopressors. To evaluate acute status discrimination, PBS examinations were assigned to the septic shock case group only if they were performed within one day of the concurrent administration of antibiotics and vasopressors. Among patients with septic shock, examinations performed during the same hospitalization episode but more than one day before or after the concurrent administration were excluded from both the case and control groups. The control group comprised the remaining examinations.

Chronic comorbidities included cardiovascular disease (CVD), chronic kidney disease (CKD), diabetes mellitus (DM), and the Charlson Comorbidity Index (CCI), all ascertained from *ICD-10* codes documented within one year before the PBS examination. For CVD (I11.0, I13.0, I13.2, I21, I22, I25.2, I25.5, I50, I60, I61, I62, I63, and I64), CKD (N18), and DM (E10, E11, E12, E13, and E14), PBS examinations were assigned to the case group if the corresponding *ICD-10* codes were present, and to the control group if no such diagnosis was documented. CCI was calculated from *ICD-10* codes and dichotomized into 2 categories: 0 to 1 versus 2 and greater [20].

### Attention-Based Cell-Type Importance

To investigate the relative importance of each cell type to model prediction for specific systemic states, we analyzed the attention weights derived from the PBS encoder in slide-level classification. For each slide, attention scores were extracted for all individual cell images, and the top 10% of cells with the highest attention scores were selected. The proportion of each cell type within these high-attention cells was calculated. These proportions were summarized as medians across all positive samples to characterize the overall distribution of high-attention cell types for specific systemic states.

### Statistical Analysis

For binary classification tasks, model performance was evaluated using the AUROC. For multiclass classification tasks, the one-versus-rest (OvR) AUROC with macro averaging was used. For cell-type classification, we assessed performance using the OvR AUROC. For all AUROC estimates, 95% CIs were computed using bootstrap resampling with 1000 iterations. To compare the AUROCs of 2 models evaluated on the same examinations, the difference in AUROC (ΔAUROC) was assessed using a paired bootstrap with 2000 iterations.

For each clinical outcome, cell-type proportions were compared between groups using the 2-sided Mann-Whitney *U* test for 2-group tasks, or the Kruskal-Wallis test followed by post hoc pairwise Mann-Whitney *U* tests for the 3-group comparison. *P* values from all comparisons were adjusted together using the Benjamini-Hochberg false discovery rate, and statistical significance was defined as an adjusted *P* value below .05.

For the exploratory longitudinal observation, a classification threshold was set at the value that maximized the Youden index on the validation set of the development dataset. This threshold was applied without reestimation to the PBS examinations in this observation.

## Results

### Dataset and Cohort Characteristics

A total of 60,580 PBS examinations comprising approximately 12 million cell images were collected (Table 1). The pretraining dataset included 51,369 PBS examinations containing approximately 10 million individual cell images. The development dataset consisted of 7520 PBS examinations from 4212 patients, and the holdout dataset consisted of 1691 PBS examinations from 1026 patients. Each PBS examination contained an average of approximately 214 total cell images, of which a mean of 152.8 (SD 27.1) and a mean of 153.1 (SD 25.3) were eligible for analysis in the development and holdout datasets, respectively. The mean age of participants was 61.3 (SD 17.1) years in the development dataset and 61.0 (SD 17.0) years in the holdout dataset, with a roughly balanced sex distribution, females comprising 46.8% (1971/4212) and 46.7% (479/1026) of the 2 datasets, respectively.

**Table 1.** Datasets and clinical characteristics.

Characteristics	Pretraining	Development		Holdout	
PBS^a^ examinations, n	51,369	7520		1691	
Total images per PBS, mean (SD)	195.4 (42.0)	214.4 (58.7)		214.3 (62.0)	
Eligible images per PBS, mean (SD)	—^b^	152.8 (27.1)		153.1 (25.3)	
Participants, n	—	4212		1026	
Age (years), mean (SD)	—	61.3 (17.1)		61.0 (17.0)	
Female, n (%)	—	1971 (46.8)		479 (46.7)	
Clinical outcomes, n (%)					
Acute inflammatory states					
WBC^c^, total	—	7241		1640	
Low	—	1757 (24.3)		357 (21.8)	
Normal	—	3883 (53.6)		863 (52.6)	
High	—	1601 (22.1)		420 (25.6)	
CRP^d^, total	—	4875		1085	
Normal	—	1669 (34.2)		368 (33.9)	
Elevated	—	3206 (65.8)		717 (66.1)	
ESR^e^, total	—	1805		380	
Normal	—	628 (34.8)		141 (37.1)	
Elevated	—	1177 (65.2)		239 (62.9)	
Septic shock, total	—	7311		1644	
Case	—	591 (8.1)		124 (7.5)	
Control	—	6720 (91.9)		1520 (92.5)	
Chronic comorbidities					
CCI^f^, high (≥2)	—	6054 (80.5)		1341 (79.3)	
CVD^g^	—	2013 (26.8)		455 (26.9)	
CKD^h^	—	1577 (21.0)		343 (20.3)	
DM^i^	—	1193 (15.9)		256 (15.1)	

a PBS: peripheral blood smear.

b Not applicable.

c WBC: white blood cell.

d CRP: C-reactive protein.

e ESR: erythrocyte sedimentation rate.

f CCI: Charlson Comorbidity Index.

g CVD: cardiovascular disease.

h CKD: chronic kidney disease.

i DM: diabetes mellitus.

The availability of laboratory test-based outcomes varied across examinations depending on the presence of corresponding laboratory test results. WBC count was available for 7241 and 1640 examinations, CRP for 4875 and 1085, ESR for 1805 and 380, and a septic shock label for 7311 and 1644 in the development and holdout datasets, respectively. The chronic comorbidity outcomes were annotated for all examinations. WBC count was low in 24.3% (1757/7241) and 21.8% (357/1640), and high in 22.1% (1601/7241) and 25.6% (420/1640); CRP was elevated in 65.8% (3206/4875) and 66.1% (717/1085); and ESR was elevated in 65.2% (1177/1805) and 62.9% (239/380) of examinations in the development and holdout datasets, respectively. Septic shock cases accounted for 8.1% (591/7311) and 7.5% (124/1644) of examinations with an available label, whereas among all examinations CVD was present in 26.8% (2013/7520) and 26.9% (455/1691), CKD in 21.0% (1577/7520) and 20.3% (343/1691), DM in 15.9% (1193/7520) and 15.1% (256/1691), and a high CCI (≥2) in 80.5% (6054/7520) and 79.3% (1341/1691), respectively. The task-specific age and sex distributions of the case and control groups for all 8 outcomes are provided in Table S1 in Multimedia Appendix 1.

### Cell-Level Representation Learning and Morphological Assessment

As the cell encoder was trained without task-specific supervision, the learned cell features are expected to encode generalizable morphology-relevant information. To validate the quality of the learned cell representations, we evaluated the cell encoder through 3 complementary approaches: (1) visualization of cell features using Uniform Manifold Approximation and Projection (UMAP) to assess cluster separability across cell types, (2) reconstruction of cell images via a diffusion model conditioned on the learned features to verify morphological fidelity, and (3) cell-type classification from cell features alone to quantify how well morphological information distinguishing cell types is captured in the representation space.

First, we applied UMAP to the cell features of 40,000 expert-annotated cells (20,000 each from the development and holdout datasets) and colored the resulting embedding by dataset (Figure 2A, left) and by cell type (Figure 2A, right). Development and holdout cells showed substantial overlap, indicating that the encoder’s representations are consistent across datasets, while cells of the same type formed distinct clusters, demonstrating clear separability across cell types. Second, to validate cell morphological feature extraction, we reconstructed cell images from the learned cell features. The reconstructions showed high fidelity to the original images, and structural characteristics of cells were preserved with only subtle variations in fine details when the stochastic subcode was varied through Gaussian sampling (Figure 3). Third, we developed a cell-type classifier to assign cell-type labels, such as neutrophil or lymphocyte, for the slide-level PBS encoder. The cell-type classifier categorized each cell into one of 9 classes. For performance evaluation, platelet and unidentifiable images were consolidated into a single noneligible class, yielding 8 classes, comprising neutrophil, lymphocyte, monocyte, eosinophil, basophil, immature cell, nRBC, and noneligible image. Cell-type classification using cell features achieved OvR AUROCs of 0.996‐1.000 for all cell types in the development and holdout datasets (Figure 2B).

**Figure 2.**
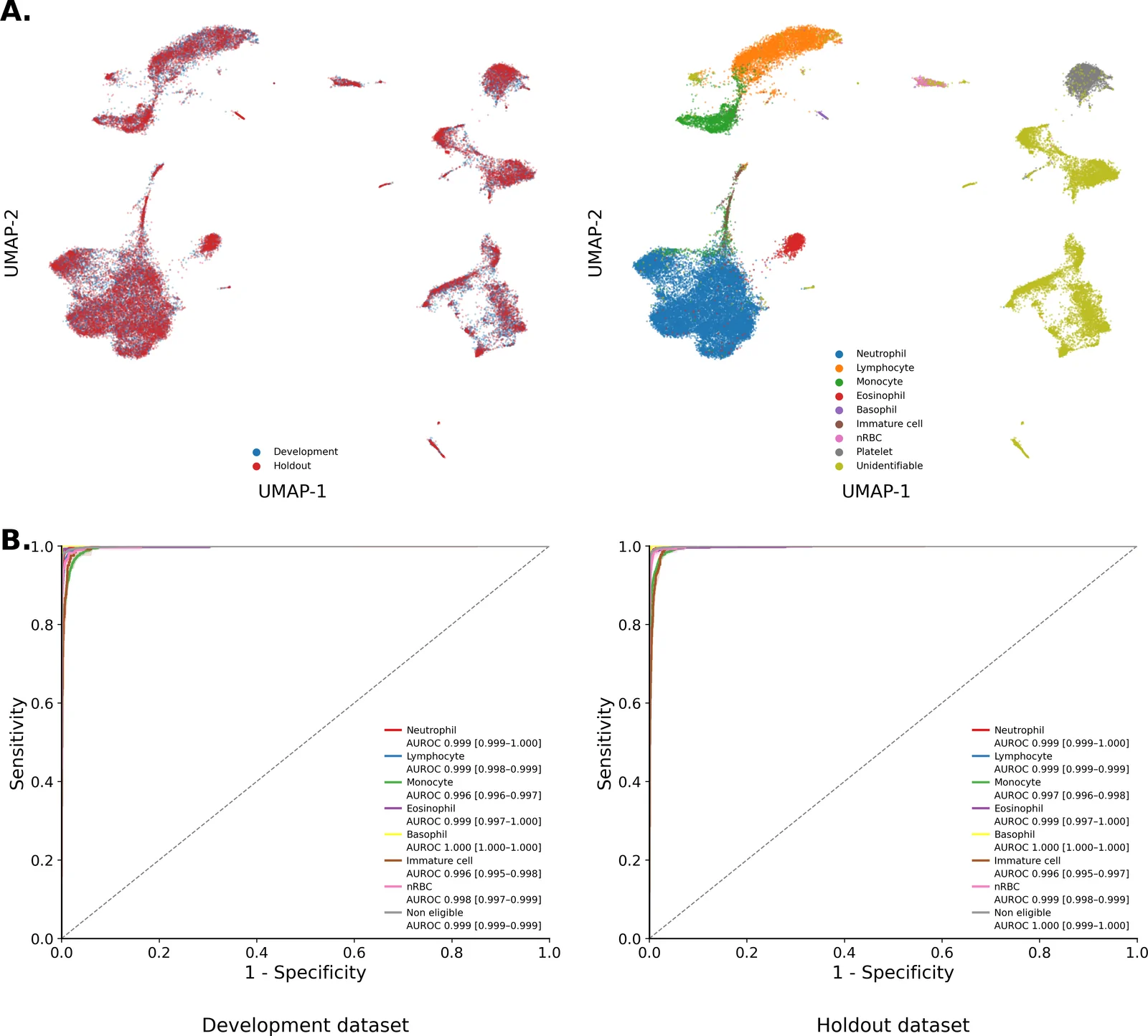
Cell-level representation learning and cell-type classification performance. A. Uniform Manifold Approximation and Projection visualization of learned cell representations from the cell encoder; the same embedding is shown colored by dataset (left) and by annotated cell type (right). B. Receiver operating characteristic curves for one-versus-rest cell-type classification in the development and holdout datasets, with the corresponding area under the receiver operating characteristic curve for each cell type. Shaded areas and bracketed values denote 95% CIs estimated by bootstrap (1000 resamples). AUROC: area under the receiver operating characteristic curve; nRBC: nucleated red blood cell; UMAP: Uniform Manifold Approximation and Projection.

**Figure 3.**
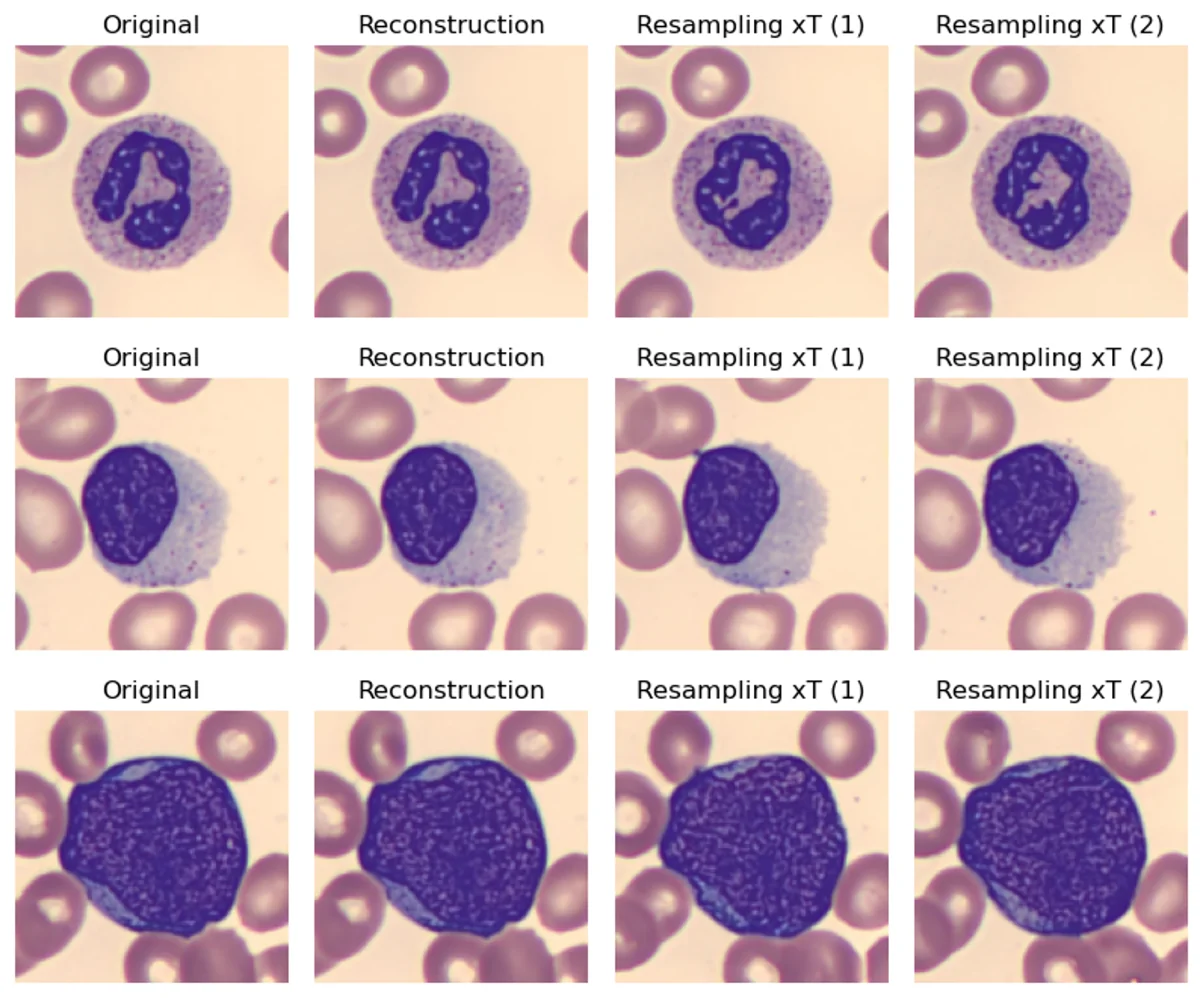
Reconstructed images with a varying subcode conditioned on fixed cell features.

### Prediction of Systemic States From Integrated Analysis of PBS Examination

We evaluated the slide-level PBS model on 8 systemic clinical targets, grouped into acute inflammatory states and chronic comorbidities (Figure 4). Across all targets, performance in the holdout cohort closely tracked that in the test set of the development cohort. For acute states, the model achieved AUROCs of 0.852 (95% CI 0.836‐0.867) and 0.849 (95% CI 0.833‐0.864) for WBC count category, 0.891 (95% CI 0.870‐0.913) and 0.896 (95% CI 0.876‐0.915) for CRP, 0.838 (95% CI 0.794‐0.878) and 0.844 (95% CI 0.799‐0.882) for ESR, and 0.849 (95% CI 0.807‐0.886) and 0.822 (95% CI 0.783‐0.853) for septic shock in the development and holdout cohorts, respectively.

**Figure 4.**
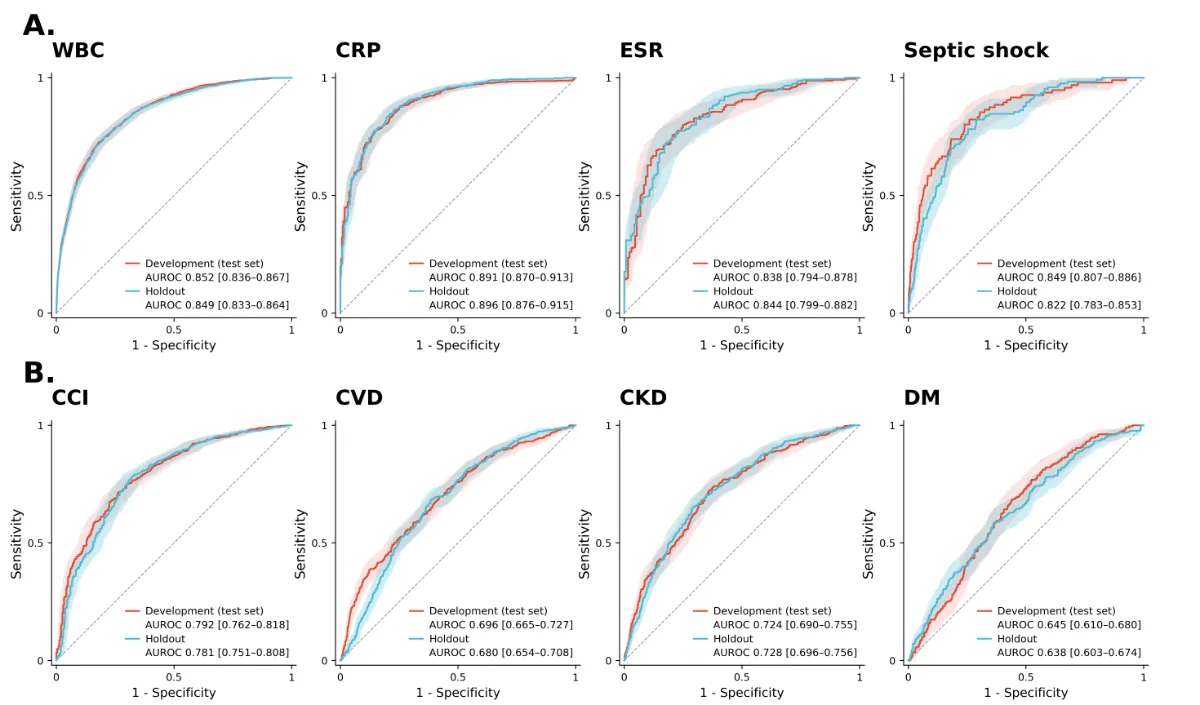
Model performance for clinical outcomes. Receiver operating characteristic curves showing model performance on the test set of the development dataset and on the holdout dataset, with the corresponding area under the receiver operating characteristic curve (AUROC) for each outcome. A. Acute inflammatory states: white blood cell count, C-reactive protein, erythrocyte sedimentation rate, and septic shock. B. Chronic comorbidities: Charlson Comorbidity Index, cardiovascular disease, chronic kidney disease, and diabetes mellitus. For white blood cell count, macro-averaged one-versus-rest AUROC is reported. Shaded areas and bracketed values denote 95% CIs estimated by bootstrap (1000 resamples). AUROC: area under the receiver operating characteristic curve; CCI: Charlson Comorbidity Index; CKD: chronic kidney disease; CRP: C-reactive protein; CVD: cardiovascular disease; DM: diabetes mellitus; ESR: erythrocyte sedimentation rate; WBC: white blood cell.

For chronic comorbidities, performance was more modest but similarly stable across cohorts, with AUROCs of 0.792 (95% CI 0.762‐0.818) and 0.781 (95% CI 0.751‐0.808) for high comorbidity burden (CCI≥2), 0.696 (95% CI 0.665‐0.727) and 0.680 (95% CI 0.654‐0.708) for CVD, 0.724 (95% CI 0.690‐0.755) and 0.728 (95% CI 0.696‐0.756) for CKD, and 0.645 (95% CI 0.610‐0.680) and 0.638 (95% CI 0.603‐0.674) for DM.

Retaining platelet images as input yielded performance comparable to that of the base model (Table S2 in Multimedia Appendix 2). Across the 8 outcomes and both datasets, the absolute difference in AUROC between the platelet-included and base models did not exceed 0.007.

### Temporal Validation

In the temporal validation, the temporal development dataset comprised 6905 PBS examinations from 4018 patients, and the temporal holdout dataset comprised 1614 examinations from 1220 patients, after 692 examinations from 337 patients who were also included in the temporal development dataset were excluded from the temporal holdout dataset (Table S3 in Multimedia Appendix 3).

Performance in the temporal holdout dataset was consistent with that in the test set of the temporal development dataset (Figure S1 in Multimedia Appendix 3). For acute states, the model achieved AUROCs of 0.846 (95% CI 0.828‐0.864) and 0.852 (95% CI 0.837‐0.867) for WBC count category, 0.889 (95% CI 0.867‐0.911) and 0.883 (95% CI 0.861‐0.903) for CRP, 0.831 (95% CI 0.784‐0.876) and 0.831 (95% CI 0.790‐0.867) for ESR, and 0.829 (95% CI 0.788‐0.865) and 0.838 (95% CI 0.800‐0.874) for septic shock in the temporal development and holdout datasets, respectively. For chronic comorbidities, AUROCs were 0.788 (95% CI 0.757‐0.818) and 0.765 (95% CI 0.737‐0.794) for high comorbidity burden (CCI≥2), 0.695 (95% CI 0.663‐0.724) and 0.689 (95% CI 0.660‐0.718) for CVD, 0.718 (95% CI 0.682‐0.752) and 0.719 (95% CI 0.690‐0.751) for CKD, and 0.666 (95% CI 0.624‐0.706) and 0.621 (95% CI 0.581‐0.660) for DM.

### Cell-Type Distribution Across Clinical Outcomes

We analyzed cell-type distributions across clinical outcomes to determine whether cell morphotypes reflect systemic states (Figure 5). For each of the 8 classification tasks, we compared the proportion of each cell type between the corresponding outcome groups. Among acute inflammatory states, septic shock showed the most pronounced shift, with a higher neutrophil proportion and a markedly lower lymphocyte proportion in cases than in controls (neutrophil 81.82% [IQR 68.67%‐90.67%] versus 66.02% [IQR 50.67%‐79.47%]; lymphocyte 3.42% [IQR 1.34%‐8.06%] versus 16.56% [IQR 7.33%‐29.46%]; monocyte 6.80% [IQR 3.31%‐13.41%] versus 7.28% [IQR 4.29%‐12.75%]). Elevated CRP showed a similar pattern (neutrophil 74.83% [IQR 57.25%‐85.76%] versus 62.50% [IQR 48.67%‐75.00%]; lymphocyte 8.00% [IQR 3.36%‐16.89%] versus 21.99% [IQR 12.24%‐33.78%]; monocyte 8.44% [IQR 4.58%‐15.72%] versus 6.71% [IQR 4.03%‐10.83%]). For WBC count, proportions shifted in a graded manner, with the neutrophil proportion decreasing progressively from the high to the normal to the low group (79.74% [IQR 57.50%‐90.00%], 68.67% [IQR 55.03%‐80.13%], and 56.29% [IQR 40.14%‐69.83%]) and the lymphocyte proportion increasing in the opposite direction (5.29% [IQR 1.99%‐13.16%], 15.20% [IQR 7.24%‐26.57%], and 23.75% [IQR 13.16%‐36.35%]). In contrast, elevated ESR showed little change, with neutrophil and lymphocyte proportions not differing significantly between the elevated and normal groups (68.98% [IQR 51.53%‐81.64%] versus 68.60% [IQR 54.34%‐82.24%] and 14.17% [IQR 6.61%‐26.59%] versus 16.11% [IQR 6.04%‐29.63%], respectively).

**Figure 5.**
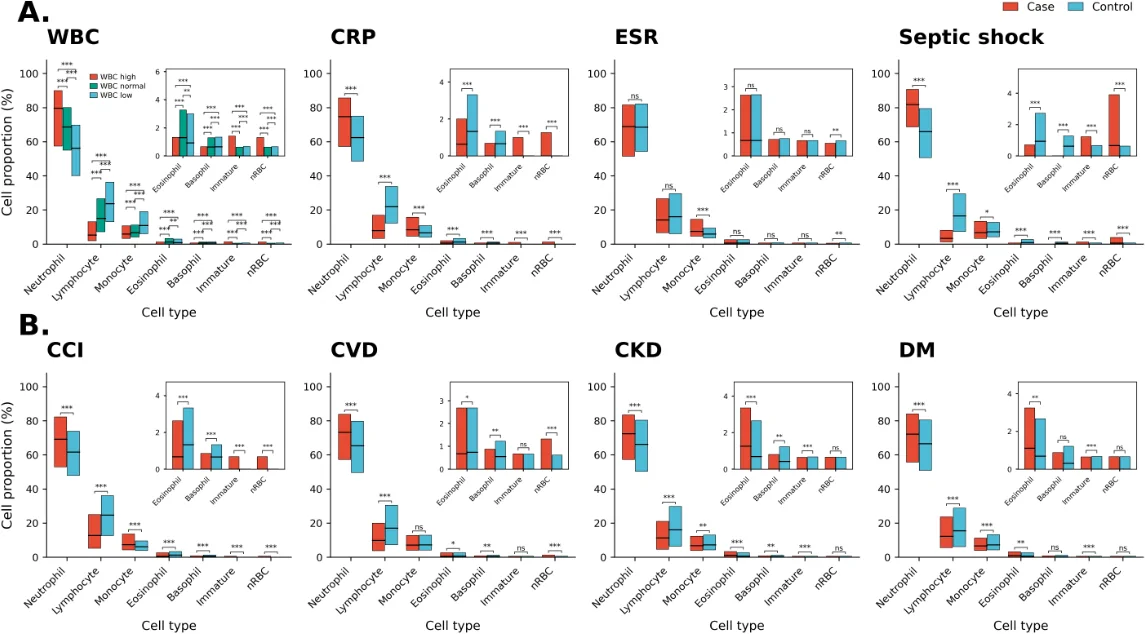
Cell proportions per cell type across clinical conditions. Distribution of cell proportions for each cell type stratified by clinical outcome. A. Acute inflammatory states: white blood cell count, C-reactive protein, erythrocyte sedimentation rate, and septic shock. B. Chronic comorbidities: Charlson Comorbidity Index, cardiovascular disease, chronic kidney disease, and diabetes mellitus. Comparisons were performed using 2-sided Mann-Whitney *U* tests for 2-group comparisons and a Kruskal-Wallis test followed by pairwise 2-sided Mann-Whitney *U* tests for the 3-group white blood cell comparison. *P* values from all comparisons were corrected together as a single family using the Benjamini-Hochberg false discovery rate; asterisks denote adjusted significance (*P<.05, **P<.01, ***P<.001), and ns denotes not significant. CCI: Charlson Comorbidity Index; CKD: chronic kidney disease; CRP: C-reactive protein; CVD: cardiovascular disease; DM: diabetes mellitus; ESR: erythrocyte sedimentation rate; nRBC: nucleated red blood cell; WBC: white blood cell.

Among chronic comorbidities, all 4 conditions showed the same directional shift, with higher neutrophil and lower lymphocyte proportions in cases than in controls. This shift was largest for CCI, in which the lymphocyte proportion in cases was 11.99 percentage points lower than in controls (12.84% [IQR 5.33%‐25.00%] versus 24.83% [IQR 12.75%‐36.14%]).

### Incremental Contribution of Cell Morphology

To assess whether the predictive performance of the framework reflected morphological information beyond cell-type composition and demographic information, we compared the base model, which uses both morphological features and cell-type embeddings, with a demographic model and a cell-type-only model (Table 2). The demographic model was a logistic regression model that used only age and sex as inputs and was trained and evaluated on the same data partitions as the base model. In the cell-type-only model, the morphological feature of each cell was omitted from its input token, and only the learnable cell-type embedding was retained, with all other components and the training and evaluation procedure held identical to those of the base model.

**Table 2.** Contribution of morphological features to slide-level prediction^a^.

Outcomes	Demographic^b^, AUROC^c^	Δ^d^	*P* value	Cell-type-only^e^, AUROC	Δ	*P* value	Base^f^, AUROC
WBC^g^							
Development (test set)	0.538 (0.517 to 0.559)	0.314 (0.287 to 0.341)	<.001	0.729 (0.709 to 0.749)	0.123 (0.103 to 0.143)	<.001	0.852 (0.836 to 0.867)
Holdout	0.534 (0.514 to 0.553)	0.315 (0.290 to 0.340)	<.001	0.731 (0.712 to 0.750)	0.118 (0.100 to 0.136)	<.001	0.849 (0.833 to 0.864)
CRP^h^							
Development (test set)	0.548 (0.509 to 0.588)	0.343 (0.300 to 0.387)	<.001	0.810 (0.780 to 0.839)	0.081 (0.056 to 0.104)	<.001	0.891 (0.870 to 0.913)
Holdout	0.552 (0.516 to 0.591)	0.344 (0.302 to 0.383)	<.001	0.813 (0.786 to 0.841)	0.083 (0.060 to 0.106)	<.001	0.896 (0.876 to 0.915)
ESR^i^							
Development (test set)	0.524 (0.462 to 0.584)	0.315 (0.242 to 0.388)	<.001	0.627 (0.567 to 0.681)	0.212 (0.148 to 0.276)	<.001	0.838 (0.794 to 0.878)
Holdout	0.618 (0.556 to 0.677)	0.226 (0.157 to 0.300)	<.001	0.600 (0.541 to 0.659)	0.244 (0.182 to 0.302)	<.001	0.844 (0.799 to 0.882)
Septic shock							
Development (test set)	0.645 (0.592 to 0.701)	0.203 (0.131 to 0.270)	<.001	0.792 (0.740 to 0.843)	0.057 (0.024 to 0.090)	<.001	0.849 (0.807 to 0.886)
Holdout	0.604 (0.550 to 0.658)	0.217 (0.158 to 0.273)	<.001	0.787 (0.748 to 0.822)	0.035 (0.008 to 0.062)	.01	0.822 (0.783 to 0.853)
CCI^j^							
Development (test set)	0.600 (0.563 to 0.639)	0.192 (0.152 to 0.229)	<.001	0.699 (0.664 to 0.733)	0.093 (0.065 to 0.121)	<.001	0.792 (0.762 to 0.818)
Holdout	0.627 (0.592 to 0.657)	0.154 (0.117 to 0.191)	<.001	0.700 (0.666 to 0.731)	0.081 (0.057 to 0.105)	<.001	0.781 (0.751 to 0.808)
CVD^k^							
Development (test set)	0.605 (0.568 to 0.638)	0.091 (0.046 to 0.138)	<.001	0.611 (0.578 to 0.645)	0.085 (0.061 to 0.111)	<.001	0.696 (0.665 to 0.727)
Holdout	0.646 (0.619 to 0.678)	0.033 (-0.006 to 0.074)	.09	0.646 (0.618 to 0.675)	0.034 (0.010 to 0.058)	.004	0.680 (0.654 to 0.708)
CKD^l^							
Development (test set)	0.600 (0.565 to 0.638)	0.124 (0.083 to 0.167)	<.001	0.600 (0.566 to 0.635)	0.124 (0.089 to 0.159)	<.001	0.724 (0.690 to 0.755)
Holdout	0.597 (0.561 to 0.631)	0.130 (0.088 to 0.173)	<.001	0.644 (0.613 to 0.675)	0.083 (0.050 to 0.116)	<.001	0.728 (0.696 to 0.756)
DM^m^							
Development (test set)	0.666 (0.630 to 0.699)	-0.021 (-0.065 to 0.024)	.34	0.494 (0.453 to 0.533)	0.151 (0.105 to 0.201)	<.001	0.645 (0.610 to 0.680)
Holdout	0.676 (0.645 to 0.706)	-0.038 (-0.078 to 0.006)	.08	0.509 (0.467 to 0.549)	0.129 (0.076 to 0.182)	<.001	0.638 (0.603 to 0.674)

a Values are area under the receiver operating characteristic curve with 95% CIs estimated by bootstrap; for white blood cell count, macro-averaged one-versus-rest area under the receiver operating characteristic curve is reported. *P* values and the 95% CIs of Δ were obtained by a paired bootstrap.

b Demographic model was a logistic regression model that used only age and sex as inputs and was trained and evaluated on the same data partitions as the base model.

c AUROC: area under the receiver operating characteristic curve.

d Δ denotes the difference in area under the receiver operating characteristic curve between the base model and each comparator model (base minus comparator).

e Cell-type-only model was trained identically to the base model but retained only the cell-type embedding.

f Base model used both cell morphological features and cell-type embeddings as input to the slide-level encoder.

g WBC: white blood cell.

h CRP: C-reactive protein.

i ESR: erythrocyte sedimentation rate.

j CCI: Charlson Comorbidity Index.

k CVD: cardiovascular disease.

l CKD: chronic kidney disease.

m DM: diabetes mellitus.

The base model achieved significantly higher AUROCs than the demographic model for 6 of the 8 outcomes in both the development and holdout cohorts (Δ 0.124‐0.344), whereas the differences were not significant for CVD in the holdout cohort, and for DM in either cohort. The base model also achieved significantly higher AUROCs than the cell-type-only model for all 8 outcomes in both the development and holdout cohorts. Nevertheless, the cell-type-only model itself retained substantial discrimination for several outcomes, with development and holdout AUROCs of 0.810 and 0.813 for CRP, 0.792 and 0.787 for septic shock, 0.699 and 0.700 for CCI, and 0.611 and 0.646 for CVD. Among acute inflammatory states, the gain in AUROC over the cell-type-only model was largest for ESR (Δ 0.212 and 0.244 in the development and holdout cohorts, respectively), followed by WBC count (Δ 0.123 and 0.118), CRP (Δ 0.081 and 0.083), and septic shock (Δ 0.057 and 0.035). Among chronic comorbidities, the gain was largest for DM (Δ 0.151 and 0.129), followed by CKD (Δ 0.124 and 0.083), CCI (Δ 0.093 and 0.081), and CVD (Δ 0.085 and 0.034). DM was the only outcome for which the cell-type-only model performed at chance level (AUROC 0.494 and 0.509), and although the base model achieved AUROCs of 0.645 and 0.638, these did not exceed those of the demographic model (0.666 and 0.676).

### Attention-Based Cell-Type Importance for Model Prediction

Lymphocytes dominated the attended cells across most acute states (Figure 6). In WBC low, lymphocytes were strongly predominant (78.6% [IQR 56.2%‐93.3%] in development, 80.0% [IQR 60.0%‐93.3%] in holdout), exceeding the next most frequent type (monocytes) by more than 60 percentage points. In WBC high, lymphocytes remained the most attended type but with a markedly reduced proportion (26.7% [IQR 11.8%‐60.0%] and 26.7% [IQR 12.5%‐60.0%]), comparable to neutrophils (25.0% [IQR 6.2%‐53.3%] and 23.5% [IQR 5.6%‐53.3%]). CRP and ESR showed nearly identical profiles, with lymphocytes most frequent (CRP 60.0% [IQR 31.2%‐86.7%] and 57.9% [IQR 27.3%‐85.7%]; ESR 60.0% [IQR 37.5%‐80.0%] and 60.0% [IQR 33.3%‐80.0%]), followed by monocytes. Septic shock showed codominance of lymphocytes (31.2% [IQR 13.3%‐62.5%] and 35.3% [IQR 19.7%‐76.6%]) and monocytes (30.8% [IQR 13.3%‐46.7%] and 25.8% [IQR 6.7%‐46.8%]), with a comparatively low neutrophil contribution (6.2% [IQR 0%‐33.3%] and 6.1% [IQR 0%‐33.1%]). Development and holdout estimates were highly concordant across all states.

**Figure 6.**
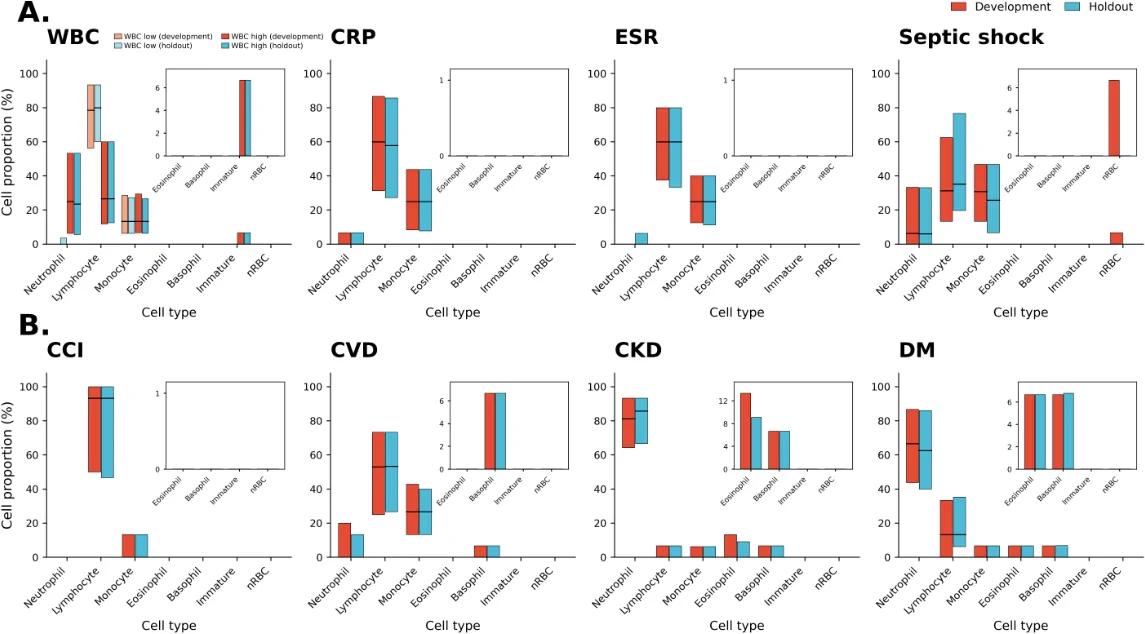
Attention-based cell-type proportion analysis. The proportion of each cell type within the top 10% most highly attended cells is shown for 8 classification tasks. A. Acute inflammatory states: white blood cell count, C-reactive protein, erythrocyte sedimentation rate, and septic shock. B. Chronic comorbidities: Charlson Comorbidity Index, cardiovascular disease, chronic kidney disease, and diabetes mellitus. CCI: Charlson Comorbidity Index; CKD: chronic kidney disease; CRP: C-reactive protein; CVD: cardiovascular disease; DM: diabetes mellitus; ESR: erythrocyte sedimentation rate; nRBC: nucleated red blood cell; WBC: white blood cell.

Among the chronic comorbidities, the most-attended cell types differed by condition rather than following a single pattern. CKD and DM were neutrophil-predominant (CKD 81.2% [IQR 64.3%‐93.3%] in development, 85.7% [IQR 66.7%‐93.3%] in holdout; DM 66.7% [IQR 43.8%‐86.7%] and 62.5% [IQR 40.0%‐86.0%]), with lymphocytes largely absent from the top-attention cells in CKD (0% [IQR 0%‐6.7%] and 0% [IQR 0%‐6.7%]). In contrast, CCI and CVD were lymphocyte-predominant (CCI 93.3% [IQR 50.0%‐100.0%] and 93.3% [IQR 46.7%‐100.0%]; CVD 52.9% [IQR 25.0%‐73.3%] and 53.3% [IQR 26.7%‐73.3%]), with neutrophils essentially absent (CCI 0% [IQR 0%‐0%] and 0% [IQR 0%‐0%]; CVD 0% [IQR 0%‐20.0%] and 0% [IQR 0%‐13.3%]). Development and holdout estimates remained highly concordant.

### Exploratory Longitudinal Observation of Morphological Changes in Septic Shock

To illustrate the prediction results of the PBS encoder through morphological changes in cells, we examined longitudinal PBS examinations in a patient with septic shock who was assigned to the test set of the development dataset; none of these examinations were used for training or validation. Septic shock probability predictions from PBS examinations were obtained at 5 time points during the clinical course of the patient (Figure 7). Based on the threshold determined by the Youden index, the PBS model correctly classified all 5 PBS examinations, comprising 2 without septic shock (nonseptic shock) and 3 with septic shock (day 1, day 2, and day 4, where day 1 denotes the first day on which both antibiotics and vasopressors were administered). Concurrently, cell images from PBS examinations with septic shock revealed morphological alterations compared with those without septic shock, with prominent changes such as cytoplasmic vacuolation in neutrophils and monocytes.

**Figure 7.**
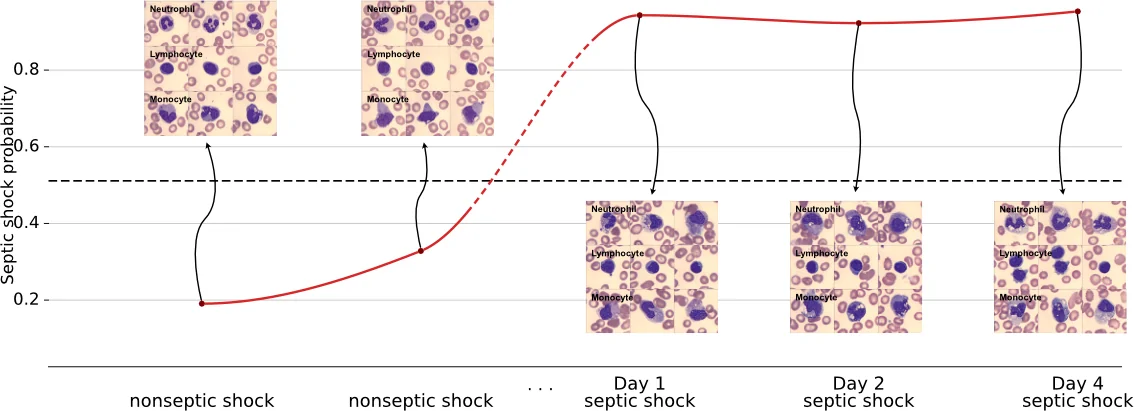
Longitudinal changes in cell morphology and corresponding model predictions. Representative cell images of neutrophils, lymphocytes, and monocytes from each examination are shown to illustrate corresponding morphological characteristics. The x-axis represents the actual clinical diagnosis at each examination, while the y-axis shows the predicted septic shock probability. The dashed line indicates the threshold determined by the Youden index on the validation set of the development dataset. Day 1 denotes the first day on which both antibiotics and vasopressors were administered.

## Discussion

### Principal Findings

In this study, we present an integrated framework that leverages morphological analysis of WBCs from PBS examinations performed in routine clinical practice to assess acute inflammatory states and chronic comorbidities. With the increasing volume of PBS examinations in clinical practice, AI-assisted PBS analysis has emerged as a promising strategy to improve the efficiency and accuracy of interpretation, which has traditionally relied on manual assessment subject to interobserver variability [21,22]. Deep learning-based approaches have been broadly explored for morphological analysis of WBCs across diverse clinical tasks, with accumulating evidence demonstrating clinical utility in hematological malignancies [14-17,23,24]. Morphological characteristics in inflammatory states are well recognized across major WBC lineages, including neutrophils, lymphocytes, and monocytes, which exhibit alterations in cell size and cellular structures during the inflammatory response [4,25,26]. In addition, morphological alterations in WBCs have been documented in PBS examinations across a wide range of infectious and inflammatory conditions [10,27-29]. Although morphological characteristics of WBCs have been widely recognized to reflect inflammatory states, the applicability of deep learning-based PBS analysis to a broader spectrum of inflammatory states beyond hematological malignancy remains largely unexplored, with only a limited number of studies investigating infectious conditions [18]. To our knowledge, this is the first study to apply deep learning-based PBS analysis to a broad range of systemic states, from acute inflammatory states to chronic comorbidities. The proposed framework showed discriminative performance for inflammation-related laboratory results and clinical outcomes by leveraging morphological characteristics from individual cell images in PBS examinations. The framework demonstrated strong performance, particularly for acute outcomes, not only for laboratory tests but also for septic shock, a complex clinical condition. This performance significantly exceeded that of the demographic model, which used age and sex only, for all 4 acute outcomes and for CCI and CKD in both cohorts (Table 2), indicating that it is not explained solely by demographic differences between the outcome groups. In a secondary temporal validation, the AUROC for each of the 8 outcomes was comparable to that in the holdout dataset of the primary analysis, and performance remained stable between the test set of the temporal development dataset and the temporal holdout dataset of previously unseen patients (Figure S1 in Multimedia Appendix 3). This consistency implies that the observed performance was not specific to the particular composition of the primary holdout dataset and was not confined to the period in which the model was developed. These findings suggest the potential of PBS examination to provide information relevant to systemic states in specific clinical contexts.

Self-supervised pretraining on large-scale cell images can yield generalizable cell-level representations [30,31]. In our framework, the cell encoder adopts a diffusion autoencoder, which can capture semantic representations of images [19], and is pretrained on approximately 10 million single-cell images derived from routine PBS examinations. The pretrained encoder effectively captures morphological characteristics of WBCs, achieving high performance in cell-type classification and producing well-separated clusters in the embedding space that correspond to distinct WBC subtypes. Furthermore, reconstruction of cell images from the learned cell features preserves the semantic structures of the original cell images, confirming that the encoder retains morphological information within its cell features.

Cell-type ratio-based indices, such as the neutrophil-to-lymphocyte ratio (NLR), have been extensively validated as biomarkers of sepsis [32-34]. The shifts in cell-type distribution observed in acute inflammatory states show that composition-based inflammatory signals can be recovered from PBS image analysis. Specifically, the septic shock group exhibited significantly increased neutrophil proportions and decreased lymphocyte proportions compared with controls. This pattern extended to the WBC high group, which similarly demonstrated an elevated neutrophil proportion and a reduced lymphocyte proportion. The observation that both acute inflammatory categories independently reproduce the proportional shift corresponding to elevated NLR is consistent with the well-established ratio-based indices. Because leukocyte differential counts were not extracted in this study, NLR or differential proportions derived from the concurrent CBC could not be computed, and a direct comparison of the framework against these indices was not performed. Instead, the cell-type-only model, which relies solely on the predicted cell-type composition of each examination, served as the composition-based comparator. In the comparison with the cell-type-only model (Table 2), the base model achieved higher discrimination for every outcome in both the development and holdout cohorts. Our analysis therefore indicates an incremental contribution of image-derived cell features beyond cell-type composition. Consistent with this interpretation, the exploratory longitudinal observation in septic shock showed morphological alterations accompanying the model predictions, such as cytoplasmic vacuolation, that are not captured by cell-type composition.

Among the acute outcomes evaluated in this study, WBC count, CRP, and ESR are not in themselves well-defined inflammatory phenotypes, but rather laboratory indices that reflect clinical conditions such as acute infection or systemic inflammation and are routinely used in clinical practice to assess systemic inflammatory states. For WBC count in particular, its value has typically already been obtained from an automated hematology analyzer by the time most PBS examinations are performed. Predicting the WBC count category (low, normal, or high) from PBS image-derived cell representations therefore serves as a way to verify how reliably the framework captures inflammation-related information. Because these indices have a ground truth that is objectively and quantitatively defined by automated hematology analyzers or standard laboratory assays, they are well suited to this assessment. That the framework discriminated these indices using PBS image-derived cell representations suggests that the learned representations encode information associated with these established clinical indices. Accordingly, these results can be interpreted as supporting the potential of PBS image analysis to be used in assessing systemic inflammatory states.

Because this study was retrospective, septic shock was not defined by clinician-adjudicated Sepsis-3 criteria but by an electronic health record (EHR)-based definition combining relevant ICD-10 codes (A40, A41, and R57.2), bacterial culture testing, and the concurrent administration of antibiotics and vasopressors. Examinations that met this operational definition and were performed within one day of antibiotic and vasopressor administration were assigned to the case group, whereas examinations that met the definition but were temporally distant from treatment were excluded from the analysis. This design was intended to remove heterogeneous examinations that are difficult to classify strictly from EHR data and to reduce misclassification. Even so, the control group may still include sepsis-related conditions that were not captured by the operational definition, so that a severely affected case group is contrasted with a clinically heterogeneous control group. This stringent case definition, together with such control heterogeneity, may have inflated the apparent discriminative performance for this outcome.

The framework demonstrated limited classification performance for chronic comorbidities, including CVD, CKD, and DM. WBCs are increasingly recognized as major mediators of chronic inflammation, contributing to sustained tissue inflammatory responses through dysregulated effector functions and impaired resolution that prolongs low-grade systemic inflammation [35-37]. Nevertheless, unlike acute inflammatory states that elicit pronounced and stereotyped morphological responses, chronic comorbidities may not induce sufficiently detectable morphological alterations in circulating blood cells, which may account for the limited classification performance observed in our framework. This was most evident for DM, for which the base model did not outperform the demographic model using age and sex only (Table 2), so the contribution of PBS image-derived cell representations should not be interpreted as a DM-specific morphological signal. In addition, the operational definition of the chronic comorbidity labels may have contributed to the modest performance. Cases for chronic comorbidities were assigned only from *ICD-10* codes documented within one year before the PBS examination. Because coding during acute admissions tends to prioritize the presenting acute problem, stable or well-managed chronic conditions are prone to under-coding, and patients with such under-recorded conditions would be misclassified into the control group. This label misclassification blurs the distinction between the case and control groups and would attenuate the apparent discriminative performance, particularly for chronic states whose morphological signal is already subtle.

### Limitations

First, the framework was developed and validated using data collected from a single center. PBS images may exhibit different characteristics depending on the slide preparation process and the imaging equipment used. Furthermore, because the composition of patient populations and the clinical pipeline through which PBS examinations are generated differ across institutions, the clinical distribution reflected by PBS examinations may itself vary from center to center. Given that both the characteristics of the data and the clinical distribution may differ across institutions, the generalizability of our findings to diverse clinical settings is limited. In addition, although a secondary temporal validation showed comparable performance, the temporal holdout period covered only 3 months, so seasonal variation could not be assessed. Second, although the pretraining and slide-level datasets (development and holdout) share no PBS examinations, patient-level overlap between them cannot be excluded, because the pretraining dataset was collected at the level of individual cell images without patient identifiers or any clinical data. Third, CRP and ESR were measured selectively only when an inflammatory state was clinically suspected. Because the analysis included only examinations with available test results, the control group, comprising patients whose test values were classified as normal, also consisted of patients in whom inflammation had been clinically suspected. Consequently, the potential for spectrum bias arising from this control group composition cannot be entirely excluded, and the model’s generalizability to general populations remains limited. Fourth, all PBS examinations were analyzed as independent samples. Because some patients underwent multiple PBS examinations, within-patient correlation was not accounted for in the cell-type proportion analysis, and the statistical significance of these comparisons may therefore be overstated. Fifth, the cell-type information utilized for noneligible image filtering and as input to the PBS encoder was derived from the output of the cell-type classifier rather than from expert-annotated labels. Although the classifier was trained on 82,000 and evaluated on 40,000 expert-annotated images, inherent error may propagate through the downstream pipeline. Moreover, because these ground-truth annotations were provided by a single laboratory physician, the potential for subjective bias in the reference labels cannot be excluded. Sixth, detailed evaluation of established morphological attributes, such as toxic granulation and cytoplasmic vacuolation, was not performed. Our framework focused on automated cell-level feature extraction through deep learning encoders rather than explicit quantification of individual morphological abnormalities. Nevertheless, longitudinal PBS examinations from a patient with septic shock showed morphological alterations, including prominent cytoplasmic vacuolation in neutrophils and monocytes, which are consistent with well-established indicators of acute inflammatory states. These observations suggest that the cell encoder may implicitly capture such morphological attributes as part of its learned cell features, even without explicit annotation.

### Conclusions

In this single-center retrospective study, integrated analysis of morphological features from PBS examination discriminated a range of inflammation-related laboratory test results and systemic states, with strong performance for acute states and more modest performance for chronic comorbidities. Our results, although exploratory, suggest the potential for deep learning-based PBS analysis to offer a novel approach to assessing systemic states in clinical practice.

## Supplementary material

10.2196/100445 Multimedia Appendix 1 Task-specific age and sex distributions across the label groups of the 8 classification tasks.

10.2196/100445 Multimedia Appendix 2 Comparison of slide-level prediction performance with and without platelet images.

10.2196/100445 Multimedia Appendix 3 Datasets, clinical characteristics, and model performance in the temporal validation.

## Abbreviations

AUROC: area under the receiver operating characteristic curve
CBC: complete blood count
CCI: Charlson Comorbidity Index
CKD: chronic kidney disease
CRP: C-reactive protein
CVD: cardiovascular disease
DM: diabetes mellitus
EHR: electronic health record
ESR: erythrocyte sedimentation rate
*ICD-10*: *International Statistical Classification of Diseases, Tenth Revision*
IRB: Institutional Review Board
NLR: neutrophil-to-lymphocyte ratio
nRBC: nucleated red blood cell
OvR: one-versus-rest
PBS: peripheral blood smear
UMAP: Uniform Manifold Approximation and Projection
WBC: white blood cell

## References

[1] Palmer L, Briggs C, McFadden S (2015). ICSH recommendations for the standardization of nomenclature and grading of peripheral blood cell morphological features. Int J Lab Hematol.

[2] Gulati G, Song J, Florea AD, Gong J (2013). Purpose and criteria for blood smear scan, blood smear examination, and blood smear review. Ann Lab Med.

[3] Leung E, Johnston A, Olsen B (2021). Laboratory practices for manual blood film review: results of an IQMH patterns of practice survey. Int J Lab Hematol.

[4] Carvalho LO, Aquino EN, Neves ACD, Fontes W (2015). The neutrophil nucleus and its role in neutrophilic function. J Cell Biochem.

[5] Radzyukevich YV, Kosyakova NI, Prokhorenko IR (2021). Participation of monocyte subpopulations in progression of experimental endotoxemia (EE) and systemic inflammation. J Immunol Res.

[6] Lam SW, Leenen LPH, van Solinge WW, Hietbrink F, Huisman A (2012). Comparison between the prognostic value of the white blood cell differential count and morphological parameters of neutrophils and lymphocytes in severely injured patients for 7-day in-hospital mortality. Biomarkers.

[7] Crouser ED, Parrillo JE, Seymour CW (2019). Monocyte distribution width: a novel indicator of sepsis-2 and sepsis-3 in high-risk emergency department patients. Crit Care Med.

[8] Zhang W, Zhang Z, Pan S (2021). The clinical value of hematological neutrophil and monocyte parameters in the diagnosis and identification of sepsis. Ann Transl Med.

[9] Sugihara J, Shibata S, Doi M (2021). Atypical lymphocytes in the peripheral blood of COVID-19 patients: a prognostic factor for the clinical course of COVID-19. PLoS One.

[10] Feng P, He Y, Guan P (2024). Serum procalcitonin, hematology parameters, and cell morphology in multiple clinical conditions and sepsis. J Clin Lab Anal.

[11] Mare TA, Treacher DF, Shankar-Hari M (2015). The diagnostic and prognostic significance of monitoring blood levels of immature neutrophils in patients with systemic inflammation. Crit Care.

[12] Asghar R, Kumar S, Shaukat A, Hynds P (2024). Classification of white blood cells (leucocytes) from blood smear imagery using machine and deep learning models: a global scoping review. PLoS One.

[13] Margret IN, Rajakumar K (2025). Deep learning techniques for analyzing peripheral blood smears: a meta-analysis. Neural Comput Applic.

[14] Eckardt JN, Middeke JM, Riechert S (2022). Deep learning detects acute myeloid leukemia and predicts NPM1 mutation status from bone marrow smears. Leukemia.

[15] Hehr M, Sadafi A, Matek C (2023). Explainable AI identifies diagnostic cells of genetic AML subtypes. PLOS Digit Health.

[16] de Almeida JG, Gudgin E, Besser M (2023). Computational analysis of peripheral blood smears detects disease-associated cytomorphologies. Nat Commun.

[17] Wei BH, Tsai XCH, Sun KJ (2025). Annotation-free deep learning for predicting gene mutations from whole slide images of acute myeloid leukemia. NPJ Precis Oncol.

[18] Cooke CL, Kim K, Xu S (2022). A multiple instance learning approach for detecting COVID-19 in peripheral blood smears. PLOS Digit Health.

[19] Preechakul K, Chatthee N, Wizadwongsa S, Suwajanakorn S Diffusion autoencoders: toward a meaningful and decodable representation.

[20] Quan H, Sundararajan V, Halfon P (2005). Coding algorithms for defining comorbidities in ICD-9-CM and ICD-10 administrative data. Med Care.

[21] Xing Y, Liu X, Dai J (2023). Artificial intelligence of digital morphology analyzers improves the efficiency of manual leukocyte differentiation of peripheral blood. BMC Med Inform Decis Mak.

[22] Wang SX, Huang ZF, Li J, Wu Y, Du J, Li T (2024). Optimization of diagnosis and treatment of hematological diseases via artificial intelligence. Front Med (Lausanne).

[23] Ouyang N, Wang W, Ma L (2021). Diagnosing acute promyelocytic leukemia by using convolutional neural network. Clin Chim Acta.

[24] Wang M, Dong C, Gao Y, Li J, Han M, Wang L (2022). A deep learning model for the automatic recognition of aplastic anemia, myelodysplastic syndromes, and acute myeloid leukemia based on bone marrow smear. Front Oncol.

[25] Shen C, Mulder E, Buitenwerf W (2023). Nuclear segmentation facilitates neutrophil migration. J Cell Sci.

[26] Simon MW (2003). The atypical lymphocyte. Int Pediatr.

[27] Sato S (2025). Recognize the significance of detecting toxic granules in sepsis. Juntendo Med J.

[28] Bozkurt M, Kuvat SV, Kapı E, Karakol P, Ozel A, Baykan H (2011). The association between cellular morphological changes in peripheral blood smear and complications in pediatric burn cases. Ulus Travma Acil Cerrahi Derg.

[29] Chander S, Sharma R, Sharma A, Verma P (2023). Toxic granules in neutrophils in sepsis patients: does it really helpful?. Muller J Med Sci Res.

[30] Koch V, Wagner SJ, Kazeminia S, Linguraru MG, Dou Q, Feragen A Medical Image Computing and Computer Assisted Intervention – MICCAI 2024: 27th International Conference, Marrakesh, Morocco, October 6–10, 2024, Proceedings, Part XII.

[31] Wenderoth L, Asemissen AM, Modemann F, Nielsen M, Werner R (2025). Transferable automatic hematological cell classification: overcoming data limitations with self-supervised learning. Comput Methods Programs Biomed.

[32] Liu X, Shen Y, Wang H, Ge Q, Fei A, Pan S (2016). Prognostic significance of neutrophil-to-lymphocyte ratio in patients with sepsis: a prospective observational study. Mediators Inflamm.

[33] Ni J, Wang H, Li Y, Shu Y, Liu Y (2019). Neutrophil to lymphocyte ratio (NLR) as a prognostic marker for in-hospital mortality of patients with sepsis: a secondary analysis based on a single-center, retrospective, cohort study. Medicine (Baltimore).

[34] Hou SK, Lin HA, Chen SC, Lin CF, Lin SF (2021). Monocyte distribution width, neutrophil-to-lymphocyte ratio, and platelet-to-lymphocyte ratio improves early prediction for sepsis at the emergency. J Pers Med.

[35] Oishi Y, Manabe I (2016). Macrophages in age-related chronic inflammatory diseases. NPJ Aging Mech Dis.

[36] Li X, Li C, Zhang W, Wang Y, Qian P, Huang H (2023). Inflammation and aging: signaling pathways and intervention therapies. Sig Transduct Target Ther.

[37] Abdullah GA, Akpan A, Phelan MM, Wright HL (2025). The complex role of neutrophils in healthy aging, inflammaging, and frailty. J Leukoc Biol.

[38] 26DeepLearningAnalysisPeripheralBloodSmear. GitHub.

