# Supplementary material for: Deep-Learning Analysis of Peripheral Blood Smear for Prediction of Acute Inflammatory States and Chronic Comorbidities: Development and Internal Validation Study
Source: JMIR Med Inform. 2026 Nov 13;14:e100445. doi: 10.2196/100445 (PMC13762691; doi:10.2196/100445)
Supplement: Supplementary file 1 [file medinform-v14-e100445-s001.docx]

**Table S1.** Task-specific age and sex distributions across the label groups of the eight classification tasks.

| Acute inflammatory states | WBC^b^ | | | | CRP^c^ | | | ESR^d^ | | | Septic shock | | |
| --- | --- | --- | --- | --- | --- | --- | --- | --- | --- | --- | --- | --- | --- |
|  | Low | Normal | High | P | Normal | Elevated | P | Normal | Elevated | P | Control | Case | P |
| PBS^a^ examinations, n | 2,114 | 4,746 | 2,021 |  | 2,037 | 3,923 |  | 769 | 1,416 |  | 8,240 | 715 |  |
| Age (years), mean±SD | 58.6±15.6 | 61.0±17.5 | 62.2±17.2 | <.001 | 59.9±16.9 | 62.0±16.1 | <.001 | 58.9±17.9 | 62.2±16.4 | <.001 | 60.4±17.2 | 65.1±16.2 | <.001 |
| Female, n (%) | 1,048 (49.6) | 2,085 (43.9) | 895 (44.3) | <.001 | 999 (49.0) | 1,674 (42.7) | <.001 | 392 (51.0) | 714 (50.4) | .81 | 3,696 (44.9) | 369 (51.6) | <.001 |

| Chronic comorbidities | CCI^e^, high (≥2) | | | CVD^f^ | | | CKD^g^ | | | DM^h^ | | |
| --- | --- | --- | --- | --- | --- | --- | --- | --- | --- | --- | --- | --- |
|  | Low | High | P | Control | Case | P | Control | Case | P | Control | Case | P |
| PBS^a^ examinations, n | 1,816 | 7,395 |  | 6,743 | 2,468 |  | 7,291 | 1,920 |  | 7,762 | 1,449 |  |
| Age (years), mean±SD | 54.8±19.6 | 62.1±16.0 | <.001 | 58.8±16.7 | 65.9±16.9 | <.001 | 59.0±17.0 | 67.0±15.7 | <.001 | 59.1±17.3 | 69.0±12.8 | <.001 |
| Female, n (%) | 977 (53.8) | 3,205 (43.3) | <.001 | 3,212 (47.6) | 970 (39.3) | <.001 | 3,375 (46.3) | 807 (42.0) | <.001 | 3,591 (46.3) | 591 (40.8) | <.001 |

^a^PBS: peripheral blood smear. ^b^WBC: white blood cell. ^c^CRP: C-reactive protein. ^d^ESR: erythrocyte sedimentation rate. ^e^CCI: Charlson Comorbidity Index. ^f^CVD: cardiovascular disease. ^g^CKD: chronic kidney disease. ^h^DM: diabetes mellitus.

Data are from the pooled development and holdout datasets. All values are per PBS examination. Age is the age at the time of examination, and the female count is reported as the number and percentage of PBS examinations in each group, whereas the female proportion in Table 1 is calculated per patient. Age is compared by one-way analysis of variance (ANOVA) for WBC (three groups) or by Welch's t-test for the other two-group tasks. The female proportion is compared by Pearson's chi-square test.
