# Supplementary material for: Deep-Learning Analysis of Peripheral Blood Smear for Prediction of Acute Inflammatory States and Chronic Comorbidities: Development and Internal Validation Study
Source: JMIR Med Inform. 2026 Nov 13;14:e100445. doi: 10.2196/100445 (PMC13762691; doi:10.2196/100445)
Supplement: Supplementary file 2 [file medinform-v14-e100445-s002.docx]

**Table S2.** Comparison of slide-level prediction performance with and without platelet images.

|  | Development (test set) | | | Holdout | | |
| --- | --- | --- | --- | --- | --- | --- |
|  | Platelet-included^a^,  AUROC | Δ^b^ | P | Platelet-included^a^,  AUROC | Δ^b^ | P |
| WBC^c^ | 0.853 (0.837 to 0.869) | 0.001 (-0.004 to 0.006) | .70 | 0.842 (0.825 to 0.857) | -0.007 (-0.011 to -0.002) | .005 |
| CRP^d^ | 0.894 (0.873 to 0.914) | 0.002 (-0.001 to 0.006) | .20 | 0.899 (0.880 to 0.917) | 0.003 (-0.001 to 0.006) | .10 |
| ESR^e^ | 0.844 (0.800 to 0.884) | 0.006 (-0.002 to 0.013) | .13 | 0.847 (0.799 to 0.884) | 0.002 (-0.007 to 0.011) | .58 |
| Septic shock | 0.848 (0.806 to 0.886) | -0.001 (-0.005 to 0.003) | .62 | 0.818 (0.779 to 0.849) | -0.004 (-0.009 to 0.001) | .13 |
| CCI^f^ | 0.790 (0.761 to 0.816) | -0.002 (-0.009 to 0.006) | .65 | 0.781 (0.752 to 0.807) | 0.000 (-0.007 to 0.007) | .93 |
| CVD^g^ | 0.694 (0.661 to 0.725) | -0.002 (-0.009 to 0.006) | .59 | 0.685 (0.660 to 0.713) | 0.006 (-0.001 to 0.012) | .10 |
| CKD^h^ | 0.726 (0.692 to 0.758) | 0.002 (-0.008 to 0.010) | .74 | 0.722 (0.690 to 0.751) | -0.006 (-0.014 to 0.001) | .11 |
| DM^i^ | 0.650 (0.615 to 0.685) | 0.005 (-0.004 to 0.013) | .24 | 0.633 (0.596 to 0.670) | -0.006 (-0.012 to 0.002) | .13 |

^a^Platelet-included model was trained identically to the base model reported in Table 2, except that cell images predicted as platelets were retained. ^b^Δ denotes the difference in AUROC between the platelet-included and base models (platelet-included minus base). ^c^WBC: white blood cell. ^d^CRP: C-reactive protein. ^e^ESR: erythrocyte sedimentation rate. ^f^CCI: Charlson Comorbidity Index. ^g^CVD: cardiovascular disease. ^h^CKD: chronic kidney disease. ^i^DM: diabetes mellitus.

Values are AUROC with 95% confidence intervals estimated by bootstrap; for WBC count, macro-averaged one-versus-rest AUROC is reported. P values and the 95% confidence intervals of Δ were obtained by a paired bootstrap.
