# Supplementary material for: Deep-Learning Analysis of Peripheral Blood Smear for Prediction of Acute Inflammatory States and Chronic Comorbidities: Development and Internal Validation Study
Source: JMIR Med Inform. 2026 Nov 13;14:e100445. doi: 10.2196/100445 (PMC13762691; doi:10.2196/100445)
Supplement: Supplementary file 3 [file medinform-v14-e100445-s003.docx]

**Table S3.** Datasets and clinical characteristics in the temporal validation.

|  | Development | | Holdout | |
| --- | --- | --- | --- | --- |
| PBS^a^ examinations, n | 6,905 | | 1,614 | |
| Total images per PBS^a^, mean±SD | 217.8±60.0 | | 201.0±53.8 | |
| Eligible images per PBS^a^, mean±SD | 152.6±27.7 | | 153.4±23.2 | |
| Participants, n | 4,018 | | 1,220 | |
| Age (years), mean±SD | 60.9±17.1 | | 62.1±16.9 | |
| Female, n (%) | 1,850 | (46.0) | 600 | (49.2) |
| Clinical outcomes, n (%) |  |  |  |  |
| Acute inflammatory states |  |  |  |  |
| WBC^b^, total | 6,652 |  | 1,556 |  |
| Low | 1,494 | (22.5) | 360 | (23.1) |
| Normal | 3,594 | (54.0) | 858 | (55.1) |
| High | 1,564 | (23.5) | 338 | (21.7) |
| CRP^c^, total | 4,452 |  | 1,043 |  |
| Normal | 1,577 | (35.4) | 350 | (33.6) |
| Elevated | 2,875 | (64.6) | 693 | (66.4) |
| ESR^d^, total | 1,609 |  | 429 |  |
| Normal | 587 | (36.5) | 150 | (35.0) |
| Elevated | 1,022 | (63.5) | 279 | (65.0) |
| Septic shock, total | 6,724 |  | 1,573 |  |
| Case | 524 | (7.8) | 114 | (7.2) |
| Control | 6,200 | (92.2) | 1,459 | (92.8) |
| Chronic comorbidities |  |  |  |  |
| CCI^e^, high (≥2) | 5,547 | (80.3) | 1,231 | (76.3) |
| CVD^f^ | 1,851 | (26.8) | 391 | (24.2) |
| CKD^g^ | 1,454 | (21.1) | 323 | (20.0) |
| DM^h^ | 1,100 | (15.9) | 234 | (14.5) |

^a^PBS: peripheral blood smear. ^b^WBC: white blood cell. ^c^CRP: C-reactive protein. ^d^ESR: erythrocyte sedimentation rate. ^e^CCI: Charlson Comorbidity Index. ^f^CVD: cardiovascular disease. ^g^CKD: chronic kidney disease. ^h^DM: diabetes mellitus.

Development, PBS examinations received between April 2024 and September 2025; Holdout, examinations received between October 2025 and December 2025 from participants with no examination during the development period.

Figure S1. Model performance for clinical outcomes in the temporal validation. Receiver operating characteristic curves showing model performance on the test set of the temporal development dataset (April 2024 to September 2025) and on the temporal holdout dataset (October 2025 to December 2025), with the corresponding area under the receiver operating characteristic curve (AUROC) for each outcome. A. Acute inflammatory states: white blood cell (WBC) count, C-reactive protein (CRP), erythrocyte sedimentation rate (ESR), and septic shock. B. Chronic comorbidities: Charlson Comorbidity Index (CCI), cardiovascular disease (CVD), chronic kidney disease (CKD), and diabetes mellitus (DM). For WBC count, macro-averaged one-versus-rest AUROC is reported. Shaded areas and bracketed values denote 95% confidence intervals estimated by bootstrap (1,000 resamples).
